# Usability and Preliminary Effectiveness of a Preoperative mHealth App for People Undergoing Major Surgery: Pilot Randomized Controlled Trial

**DOI:** 10.2196/23402

**Published:** 2021-01-07

**Authors:** Miriam van der Velde, Karin Valkenet, Edwin Geleijn, Marjoke Kruisselbrink, Marije Marsman, Liedewij MJ Janssen, Jelle P Ruurda, Donald L van der Peet, Jesse J Aarden, Cindy Veenhof, Marike van der Leeden

**Affiliations:** 1 Innovation of Human Movement Care Research Group HU University of Applied Sciences Utrecht Netherlands; 2 Department of Rehabilitation, Physical Therapy Science and Sports University Medical Center Utrecht Utrecht University Utrecht Netherlands; 3 Department of Rehabilitation Medicine Amsterdam University Medical Center Vrije Universiteit Amsterdam Amsterdam Netherlands; 4 Clinical Health Sciences Program Physiotherapy Sciences Utrecht University Utrecht Netherlands; 5 Department of Anesthesiology University Medical Center Utrecht Utrecht University Utrecht Netherlands; 6 Department of Anesthesiology Amsterdam University Medical Center Vrije Universiteit Amsterdam Amsterdam Netherlands; 7 Department of Surgery University Medical Center Utrecht Utrecht University Utrecht Netherlands; 8 Department of Surgery Amsterdam University Medical Center Vrije Universiteit Amsterdam Amsterdam Netherlands; 9 European School of Physiotherapy Faculty of Health Amsterdam University of Applied Sciences Amsterdam Netherlands; 10 Amsterdam Public Health Research Institute Amsterdam University Medical Center Vrije Universiteit Amsterdam Amsterdam Netherlands

**Keywords:** preoperative care, smartphone, mhealth, risk behavior, prehabilitation, usability

## Abstract

**Background:**

Major surgery is associated with negative postoperative outcomes such as complications and delayed or poor recovery. Multimodal prehabilitation can help to reduce the negative effects of major surgery. Offering prehabilitation by means of mobile health (mHealth) could be an effective new approach.

**Objective:**

The objectives of this pilot study were to (1) evaluate the usability of the Be Prepared mHealth app prototype for people undergoing major surgery, (2) explore whether the app was capable of bringing about a change in risk behaviors, and (3) estimate a preliminary effect of the app on functional recovery after major surgery.

**Methods:**

A mixed-methods pilot randomized controlled trial was conducted in two Dutch academic hospitals. In total, 86 people undergoing major surgery participated. Participants in the intervention group received access to the Be Prepared app, a smartphone app using behavior change techniques to address risk behavior prior to surgery. Both groups received care as usual. Usability (System Usability Scale), change in risk behaviors 3 days prior to surgery, and functional recovery 30 days after discharge from hospital (Patient-Reported Outcomes Measurement Information System physical functioning 8-item short form) were assessed using online questionnaires. Quantitative data were analyzed using descriptive statistics, chi-square tests, and multivariable linear regression. Semistructured interviews about the usability of the app were conducted with 12 participants in the intervention group. Thematic analysis was used to analyze qualitative data.

**Results:**

Seventy-nine people—40 in the intervention group and 39 in the control group—were available for further analysis. Participants had a median age of 61 (interquartile range 51.0-68.0) years. The System Usability Scale showed that patients considered the Be Prepared app to have acceptable usability (mean 68.2 [SD 18.4]). Interviews supported the usability of the app. The major point of improvement identified was further personalization of the app. Compared with the control group, the intervention group showed an increase in self-reported physical activity and muscle strengthening activities prior to surgery. Also, 2 of 2 frequent alcohol users in the intervention group versus 1 of 9 in the control group drank less alcohol in the run-up to surgery. No difference was found in change of smoking cessation. Between-group analysis showed no meaningful differences in functional recovery after correction for baseline values (β=–2.4 [95% CI –5.9 to 1.1]).

**Conclusions:**

The Be Prepared app prototype shows potential in terms of usability and changing risk behavior prior to major surgery. No preliminary effect of the app on functional recovery was found. Points of improvement have been identified with which the app and future research can be optimized.

**Trial Registration:**

Netherlands Trial Registry NL8623; https://www.trialregister.nl/trial/8623

## Introduction

Every year, approximately 635,000 adults in the Netherlands undergo major surgery [1]. Major surgery is associated with negative postoperative outcomes such as complications and delayed or poor recovery [2]. Risk behaviors, such as smoking, excessive alcohol consumption, and physical inactivity, and risk factors like poor nutritional status may elevate the chance of poor postoperative outcomes in people undergoing major surgery [2-6].

Prehabilitation programs can be used to address these risks prior to major surgery. Prehabilitation is the process of improving an individual’s functional capacity by modifying risk behaviors to enable them to withstand the forthcoming stressor of major surgery [7]. There is increasing evidence for the effectiveness of prehabilitation in reducing the negative effects of major surgery [3,4].

As risk behaviors rarely occur alone, prehabilitation programs will often need to address multiple factors. These programs often consist of a combination of physical training (unsupervised or supervised by a physical therapist), nutritional support by a dietitian, smoking and alcohol cessation support, and/or psychological support [4,8,9]. Changing risk behaviors before major surgery offers opportunities and challenges. The preoperative period is considered a teachable moment, a useful time to facilitate change [10]. Some research indicates that hospitalization or upcoming surgery increases motivation to change risk behaviors [11]. In addition, patients scheduled for elective surgery might be more willing to change their risk behavior preoperatively given the restricted period of behavior change.

Even though the preoperative period is considered a teachable moment, it is also a stressful period for many patients, which poses a challenge in terms of changing risk behavior [12,13]. Furthermore, patients experience various barriers to participate in prehabilitation (eg, problems with transportation, finding time, and bearing costs). Offering prehabilitation in one’s own environment by means of mobile health (mHealth) could be an effective new approach, making prehabilitation easily accessible to many patients and helping to overcome experienced barriers to participation [14]. However, evidence for the use of mHealth apps for multimodal prehabilitation is lacking.

In this study, we evaluate the first version of the Be Prepared (Beter Voorbereid, in Dutch) mHealth app. The content of the app was developed by a team of health care professionals and health care researchers collaboratively with patients to optimize the process of prehabilitation and overcome barriers. The app uses behavior change techniques to address risk behaviors and enhance patients’ health prior to surgery in order to achieve a better postoperative functional recovery [15].

The Centre for eHealth Research (CeHRes) roadmap, a 5-step development, evaluation, and implementation approach, was used as a guideline during the development and evaluation of the Be Prepared app [16]. In this pilot study, we describe the first steps in the evaluation.

This evaluation focuses on the usability of the app and the first effects on functional outcomes. Usability has been identified as an essential criterion for the evaluation of digital apps in health care since poor usability can influence the use of the app and thereby affect its effectiveness [17].

Therefore, the primary aim of this pilot study is to evaluate the usability of the Be Prepared app prototype. In addition, we explore whether the app is capable of bringing about a change in risk behaviors in people undergoing major surgery, and we estimate a preliminary effect of the Be Prepared app on functional recovery after major surgery.

## Methods

### Design

This multicenter pilot randomized controlled trial (RCT) is part of the development approach following the CeHRes roadmap [16]. The first stages of the CeHRes roadmap (contextual inquiry, value specification, and design) and this pilot study provide the basis for the subsequent steps (operationalization and summative evaluation). These steps will be conducted in the next phase of this project. This pilot study was conducted to evaluate the usability and preliminary effectiveness of the Be Prepared app and feasibility of study procedures to identify points of improvement before conducting a large multicenter RCT. In this article, we focus on the evaluation of usability and preliminary effectiveness of the app.

Patients scheduled for major elective surgery were recruited from the preoperative assessment outpatient clinic of two academic hospitals in the Netherlands between November 2018 and March 2019. Patients were informed about the study by the anesthetist, anesthesiology nurse, or anesthesiology assistant during their preoperative assessment. Patients who were interested in participation received written information about the study and were called the next day by the investigator (at least 24 hours after being informed about the study). During the phone call, the patient was asked whether they wanted to participate, and inclusion criteria were checked. Eligible patients signed the informed consent form provided with the written information and sent it back to the investigators. After completing the baseline questionnaires, participants were randomly assigned to either the intervention or control group using a web-based randomization system. The intervention group received a link to the Be Prepared app via email. Both groups received care as usual (eg, verbal information and information leaflets). Quantitative data were collected at baseline, 3 days before surgery, and 30 days after hospital discharge. Patient characteristics and perioperative factors were collected via the electronic health record. All other data were collected through questionnaires completed by the participant on a secured web-based system. Qualitative data were collected pre- or postoperatively from a selection of app users. Participants could indicate on the informed consent form whether they gave permission to be approached for a telephone interview.

The medical ethical committee of the Amsterdam University Medical Center approved this study (NL61503.029.18). No changes were made to the design after the study was approved. We followed the Consolidated Standards of Reporting Trials (CONSORT) guidelines and the CONSORT EHEALTH (Electronic and Mobile Health Applications and Online Telehealth) checklist [18,19]. The main study is registered at the Netherlands Trial Registry [NL8623], but this pilot study is not.

### Participants

Patients scheduled for major elective surgery were eligible to be included if they were aged 18 years or older, had an indication for postoperative hospital stay of at least 2 nights, and had one or more risk behaviors. For the purpose of inclusion, we categorized risk behaviors into binary variables representing risk status based on the recommendations of the Dutch Health Council and evidence (ie, currently smoking, alcohol consumption 1 or more drinks every day, moderate intensity physical activity less than 30 minutes every day, muscle strengthening activities on fewer than 2 days per week, and/or unintentional weight loss of more than 3kg in the last month) [2,20-22]. Participants were excluded if they had no access to a mobile device or had an insufficient command of the Dutch language. Patient characteristics (age, gender, BMI, physical functioning, type of surgery, American Society of Anesthesiologists physical status classification, presence of risk behaviors) were collected in order to describe the sample.

### Intervention

The Be Prepared app prototype (Patient Journey platform by Interactive Studios BV) is a smartphone app which uses behavior change techniques to support patients in optimizing their health and risk behaviors prior to surgery. A behavior change technique is a strategy that helps an individual change their behavior to promote better health. Techniques like setting goals, advice on stop smoking medication, social support, feedback on behavior, and providing information on the health consequences of alcohol consumption and alcohol cessation were used in this app [15,23]. For example, participants were encouraged to exercise with a buddy and call someone when they felt the urge to smoke and were informed by a pulmonologist about the health risks of smoking before a major operation. In the app, participants answered questions about their risk behavior and received tailored information and advice based on the given answers. Current smokers were supported with smoking cessation prior to surgery. Frequent alcohol users were supported to decrease their alcohol intake. Inactive participants were supported to increase their amount of physical activity to at least 30 minutes of moderate intensity physical activity per day. Participants who did muscle strengthening activities less than twice a week were supported to increase these activities to at least twice a week in combination with increasing protein-rich food in their diet. Participants who unintentionally lost more than 3 kg during the past month were advised on protein and energy enriched food. Additionally, participants received information about preoperative fasting and the use of blood coagulation medication prior to surgery.

Participants in the intervention group received access to the Be Prepared app for use on their own mobile device. The introduction screen showed only basic information about the goal and use of the app. They could unlock the additional information and advice by entering a personal code they received via email.

**Figure 1:**
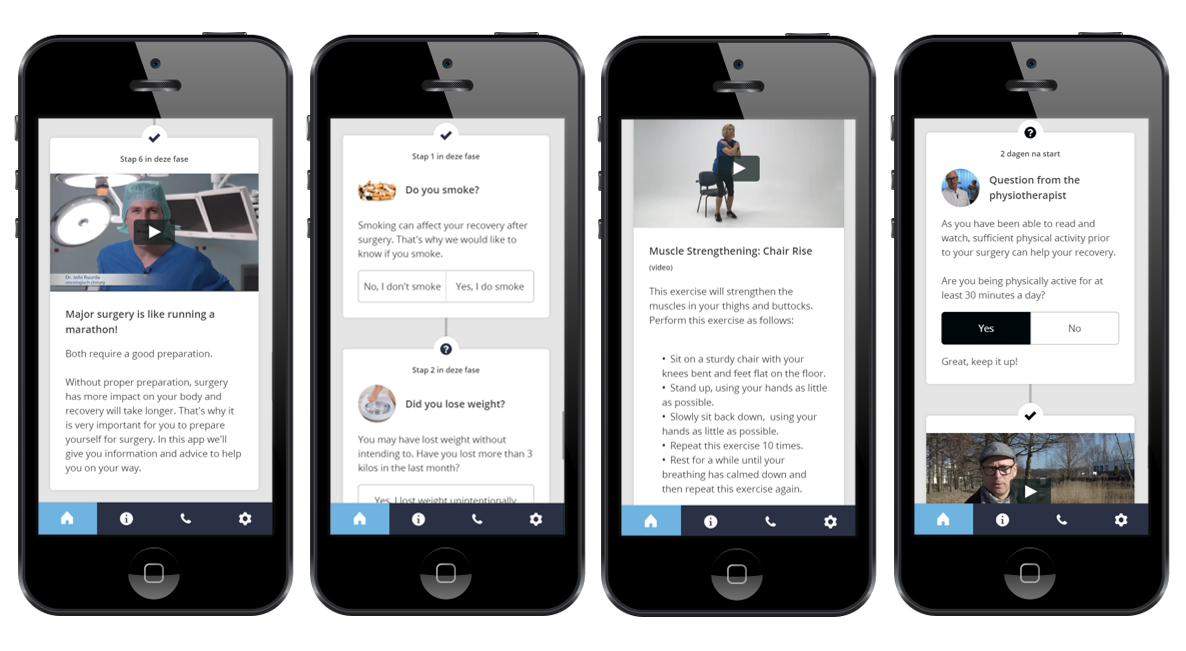
Screenshots of the Be Prepared app, translated from Dutch. From left to right, information about the purpose of the app, screening questions for app personalization, muscle strengthening exercise video, and check for progress with feedback video.

The information and advice in the app were displayed on a dynamic timeline based on the patient’s operation date. Through this dynamic timeline, day-to-day information was offered in various ways to meet different needs. The timeline provided written information and videos, tips on healthy behavior and changing risk behaviors, quizzes, and exercise videos (Figure 1). Furthermore, participants were asked whether they succeeded in following the advice and received feedback based on their response. Push notifications informed the patient about available new information and advice. This prototype version of the app provided information and advice for a maximum of 14 days prior to surgery. An overview of the app content is provided in Multimedia Appendix 1.

### Data Collection

#### Usability

Usability of the mHealth intervention was assessed both quantitatively and qualitatively. Usability is the extent to which a product can be used by specified users to achieve specified goals with regard to effectiveness, efficiency, and satisfaction [24].

##### App Use

Whether a participant in the intervention group activated the app with their personal code was logged anonymously in the database of Interactive Studios (ISO 27001 and NEN7510 certified). In the online questionnaire, participants in the intervention group were asked whether they had used the app and, if applicable, were asked about reasons for nonuse.

##### Quantitative Data

The Dutch translation of the System Usability Scale (SUS) was used to assess usability among all app users. Participants completed the SUS 3 days prior to surgery. The SUS is a reliable and valid 10-statement usability scale suitable to assess a wide range of eHealth technologies. The total SUS score ranges from 0 to 100, and higher scores reflect higher usability [25,26]. An SUS score of at least 62.7 was considered acceptable [25,27], and 68 or above was regarded as above average in terms of usability quality.

##### Qualitative Data

For the qualitative data collection, semistructured telephone interviews were conducted with a selection of participants to gain more detailed insight into the usability of the app. Interviews took 20 to 30 minutes. During the interviews, a topic list was used based on the usability components described by Nielsen [28]: efficiency, satisfaction, learnability, memorability, and tolerance for errors. The topic list is provided in Multimedia Appendix 2. Preoperative interviews were conducted 3 or more days before surgery and postoperative interviews between 1 and 2 weeks after hospital discharge. Participants were included until maximum variation in patient characteristics (age, gender, days of app use, interviewing pre- or postoperatively, and length of hospital stay) and data saturation in 3 consecutive interviews was reached.

#### Risk Behaviors

Risk behaviors were assessed through self-report at baseline and 3 days before surgery. Participants were asked to indicate on how many days per week they were physically active for at least 30 minutes, on how many days per week they performed muscle strengthening activities, and on how many days per week they consumed one or more alcoholic beverages. Participants were asked whether they were currently smoking. Current smokers were asked to indicate how many cigarettes they had smoked last week.

Participants were also asked to self-report their change in risk behaviors using a set of closed-ended questions at 3 days before surgery. All participants were asked whether they had made positive changes to their risk behaviors regarding smoking, alcohol intake, unintentional weight loss, physical activities, and/or muscle strengthening activities prior to surgery.

#### Functional Recovery

Functional recovery after surgery was assessed by the Patient-Reported Outcomes Measurement Information System physical functioning 8-item short form (PROMIS-PF) at baseline and 30 days after hospital discharge. The PROMIS-PF is derived from the Dutch PROMIS physical function item bank consisting of 121 items concerning daily activities. The short form consists of 8 questions which can be scored on a 5-point Likert scale from 1 (unable to do) to 5 (performed without any difficulty). The PROMIS-PF has a high reliability and validity and is applicable to patients with different conditions [29,30].

### Data Analysis

Descriptive statistics were used to describe patient characteristics and app use. No a priori level of statistical significance was set as this pilot study was not powered to assess effect. Point estimators and confidence intervals are given to estimate the effects, and *P* values are provided to give an impression of the evidence against the null hypothesis [31]. Complete case analyses were performed according to the intention-to-treat principle. Quantitative data were analyzed using SPSS Statistics version 25.0 (IBM Corporation).

#### Usability

##### Quantitative Data

The total SUS score was calculated using the method by Brooke [25], and descriptive statistics were used to describe scores. In order to have a better insight into the different aspects of usability, the SUS statements were subdivided into the categories learnability, efficiency, and satisfaction [32]. Mean scores per SUS statement and per category of usability were calculated using the method by Brooke [25] with the addition of a factor 10 to get a range of 0 to 100 per statement.

##### Qualitative Data

Qualitative analysis of interview data was done following the steps of thematic analysis: compiling, disassembling, reassembling, interpreting, and concluding [33-35]. Interviews were transcribed verbatim, and data were coded through open coding. Axial coding was discussed by two researchers to define definitive codes. Subsequently, codes were put into context with each other to create themes. Differences were discussed until consensus was reached. Next, analytical conclusions were made from the data presented as codes and themes. The main themes and findings regarding usability are described in the Results section as an addition to the quantitative analysis of usability.

#### Risk Behaviors

Descriptive statistics were used to describe changes in risk behaviors. Bootstrapping methods (1000 samples) were used to calculate confidence intervals for medians. Chi-square tests for linear trend were performed to examine the relation between group allocation and change scores in days of performing physical activities and muscle strengthening activities. Chi-square tests or Fisher exact tests were used to test the difference in distribution of self-reported change of all risk behaviors between allocation groups.

#### Functional Recovery

Total raw scores on the PROMIS-PF were translated into a t score for each participant using the PROMIS score conversion table [36]. The t score rescales total raw score into a standardized score with a mean of 50 and a standard deviation of 10. Descriptive statistics were used to describe physical functioning at baseline and 30 days after discharge from hospital. Bootstrapping methods were used to estimate confidence intervals for medians. A PROMIS-PF score at 30 days after discharge from hospital greater than or equal to the score at baseline was considered functionally recovered. Between-group differences in functional recovery measured by the postsurgery PROMIS-PF corrected for baseline PROMIS-PF were analyzed using multivariable linear regression.

## Results

### Flow of Participants Through the Study

**Figure 2:**
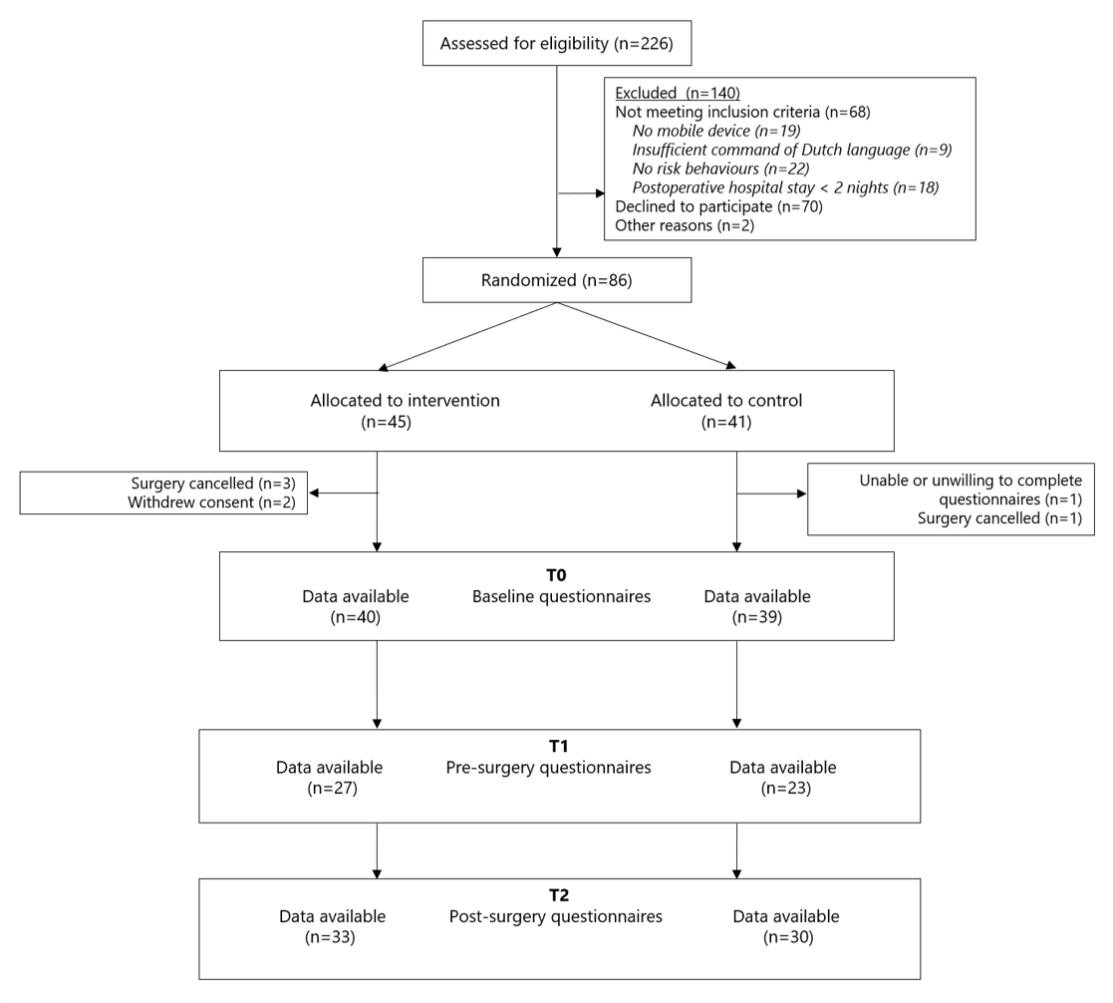
Design of study and flow of participants through the trial.

Between November 2018 and February 2019, 226 patients were screened for eligibility; 86 people were eligible and signed informed consent, with 45 participants randomized to the experimental group and 41 to the control group. In the control group, one participant did not complete baseline questionnaires and was therefore excluded from further analysis, and 4 participants were excluded from further analysis because their surgeries were cancelled. In the intervention group, 2 participants withdrew informed consent due to nursing home admission and start of palliative care. Thus, there were 40 evaluable participants in the intervention group and 39 in the control group (Figure 2).

The median age of participants was 61.0 (interquartile range [IQR] 51.0-68.0) years, 49% (39/79) were female; 34% (27/79) of participants had 1 risk behavior at baseline, 48% (38/79) had 2 risk behaviors, and 18% (14/79) had 3 or more. Of the participants, 81% (64/79) were insufficiently physically active, and the median waiting time for surgery was 28 (IQR 16-52) days. The groups were similar at baseline in terms of demographic and clinical characteristics (Table 1).

**Table 1:** Baseline characteristics of participants.

Characteristic		Total (n=79)	Intervention (n=40)	Control (n=39)
Age in years, median (IQR)		61.0 (51.0-68.0)	59.0 (43.8-64.0)	63.0 (53.0-70.0)
Female, n (%)		39 (49)	22 (55)	17 (44)
BMI (kg/m^2^), median (IQR)		25.8 (23.9-28.3)	25.5 (23.1-27.7)	26.5 (24.5-28.8)
**ASA PS^a^** **classification, n (%)**				
	I	9 (12)	7 (17)	2 (5)
	II	42 (53)	21 (53)	21 (54)
	III	24 (30)	11 (28)	13 (33)
	IV	3 (4)	1 (2)	2 (5)
	Unknown	1 (1)	0 (0)	1 (3)
**Surgical specialty, n (%)**				
	Neurosurgical	16 (20)	9 (23)	7 (18)
	Cardiothoracic	16 (20)	7 (18)	9 (23)
	Gastrointestinal	15 (19)	8 (18)	7 (18)
	Oral and maxillofacial	12 (15)	4 (10)	8 (20)
	Urologic and gynecologic	13 (17)	8 (20)	5 (13)
	Orthopedic	3 (4)	0 (0)	3 (8)
	Vascular	3 (4)	3 (8)	0 (0)
	Other	1 (1)	1 (3)	0 (0)
Waiting time for surgery in days, median (IQR)		28 (16-52)	27 (16-46)	29 (16-65)
**Risk behaviors^b^, n (%)**				
	Smoking	7 (9)	4 (10)	3 (8)
	Alcohol consumption	12 (15)	2 (5)	10 (26)
	Physical activities	64 (81)	35 (88)	29 (74)
	Muscle strengthening activities	57 (72)	29 (73)	28 (72)
	Unintentional weight loss	7 (9)	3 (8)	4 (10)
**Number of risk behaviors, n (%)**				
	1	27 (34)	12 (30)	15 (39)
	2	38 (48)	23 (58)	15 (39)
	3	12 (15)	5 (12)	7 (17)
	4	2 (2)	0 (0)	2 (5)
PROMIS-PF^c^ (t score), median (IQR)		47.8 (40.8-60.1)	47.8 (42.3-60.1)	46.7 (40.1-60.1)

^a^ASA PS: American Society of Anesthesiologists physical status.

^b^Multiple response options.

^c^PROMIS-PF: Patient-Reported Outcomes Measurement Information System physical functioning 8-item short form.

### Usability

#### App Use

Of the participants in the intervention group, 73% (29/40) activated the app with their personal code. Reasons for nonuse were unable to access the app (2/29) and not seeing added value of app use (1/29). For the other participants, reasons for nonuse are unknown. There were no apparent differences in characteristics between app users and nonusers.

#### Quantitative Results

**Figure 3:**
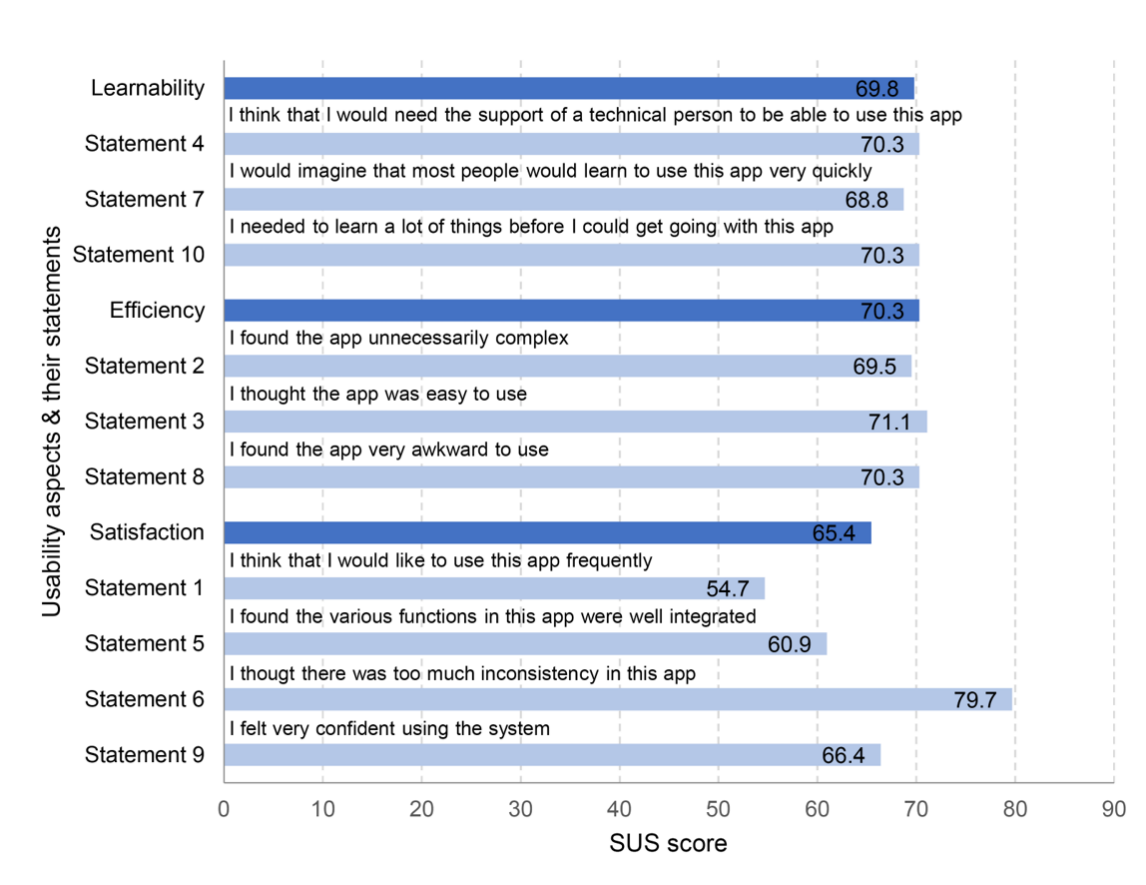
System Usability Scale scores per statement and usability aspect (higher scores reflect higher usability).

The SUS was completed by 80% (32/40) of participants from the intervention group. The usability of the Be Prepared app scored 68.2 (SD 18.4). The mean SUS scores per usability aspect were: learnability 69.8 (SD 22.5), efficiency 70.3 (SD 23.5), and satisfaction 65.4 (SD 21.9). Figure 3 shows the SUS scores per statement and usability aspect.

#### Qualitative Results

##### Interviewees

Of the participants in the intervention group, 33% (13/40) were approached for a telephone interview. One participant declined to participate because the interview would take too much time. After 12 interviews, data saturation was reached. Table 2 presents the characteristics of the interviewees. After axial coding, 5 themes were formed. The qualitative results will be described per theme.

**Table 2:** Characteristics of interviewees.

Code	Gender	Age (years)	App use (number of days)	Interview pre- or postoperative	Days of hospitalization
1	F	35	15	Pre	4
2	M	65	3	Pre	2
3	M	76	4	Pre	3
4	M	68	3	Pre	5
5	F	63	17	Post	4
6	M	49	42	Pre	Waitlisted
7	F	43	34	Post	3
8	F	59	16	Post	7
9	F	63	24	Post	7
10	M	36	22	Post	1
11	F	52	38	Pre	6
12	M	77	84	Post	7

##### Ease of Use of the App

The log-in procedure was difficult for many interviewees, and some needed help from family or the research team to log in.

I had some trouble opening the app, but then I went back to the instructions and it clearly stated what I had to do.

Male, age 65

The interviewees did not experience problems when using the app.

It is self-explanatory.

Male, age 76

##### Contents of the App Should Meet Personal Preferences

The interviewees were satisfied with the various ways in which information was presented in the app and appreciated the practical advice.

Suggestions are being made in the app that I found useful. For me it was combining walking with doing groceries. I thought that was a clever one, for me as well; if you go somewhere anyway, go walking. I even did that yesterday.

Male, age 65

The app did not match everyone’s level of functioning, and interviewees saw the need for more personalization in the app.

There are, of course, very fit people and people who have been living unhealthy for a long time. So, it will be difficult to differentiate. There could be an option menu of some sort in the app.

Male, age 65

##### App as Motivational Tool for Behavior Change

For the majority of the interviewees, getting a reminder to exercise more or eat more protein-rich food was enough to change their behavior while some others preferred advice from a health care professional.

An app is useful because you read it. Only knowing something, does not mean you will do it, but when you read it in an app you are reminded that you have to do it.

Female, age 52

The app emphasized the importance of changing risk behavior before major surgery. This motivated many interviewees. The push notifications supported most interviewees in their behavior change.

Some days I forgot or I was busy, so on those days the notifications came in handy.

Female, age 35

##### General Motivation for Behavior Change Before Major Surgery

The urgency to prepare for the upcoming surgery seemed to be the greatest motivation for the interviewees. Interviewees with a waiting time of a few weeks until their surgery felt the need to change their risk behavior.

I feel the need, I’m going into surgery next week. It has to stop raining, because this afternoon I have to work in my garden for at least 30 minutes as a physical activity.

Male, age 65

Interviewees who had to wait more than 4 weeks for surgery or who didn’t know their surgery date did not feel the urge to change until a few weeks before surgery.

The longer you have to use something like that, the greater the chance that you will not finish it.

Male, age 65

##### Views on What Constitutes a Good Preparation for Major Surgery

According to the majority of interviewees, a good preparation should benefit postoperative recovery.

So you can manage better when you get back home.

Female, age 59

Being well-informed was also mentioned as a key factor for good preparation.

To know what you can expect, what you can and cannot do. That is important, because of course you don’t know, you don’t know what’s wise to do.

Male, age 68

### Risk Behaviors

#### Physical Activities

**Figure 4:**
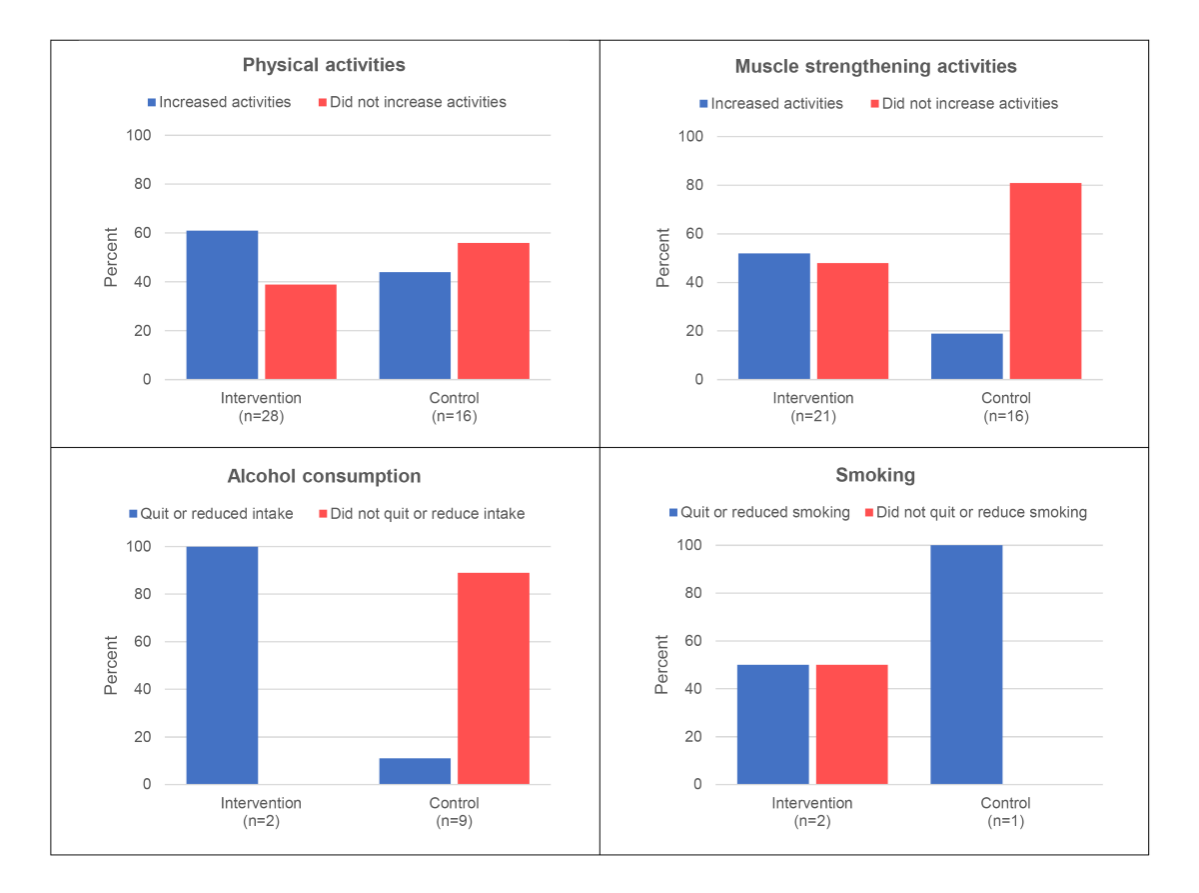
Self-reported change in risk behavior.

Of the participants, 81% (64/79) were physically inactive at baseline, of whom 69% (44/64) completed the presurgery follow-up questionnaire. At baseline, the median number of days on which participants were physically active for at least 30 minutes was 3 (95% CI 2.5 to 4.0) in the intervention group (n=28) and 4 (95% CI 2.0 to 5.0) in the control group (n=16). The intervention group became active on more days of the week after the intervention period (+1.0 day [95% CI 0.0 to +2.0]) compared with the control group (0.0 days [95% CI –0.5 to +1.0], *P*=.12). Figure 4 shows that a bigger proportion of participants in the intervention group reported having increased their physical activities prior to surgery (17/28, 61%) compared with the control group (7/16, 44%; *P*=.28).

#### Muscle Strengthening Activities

Of the participants, 72% (57/79) had risk behavior regarding muscle strengthening activities, of whom 65% (37/57) completed the presurgery follow-up questionnaire. At baseline, the median number of days on which participants performed muscle strengthening activities was 0 (95% CI 0.0 to 0.0) in both the intervention (n=21) and control group (n=16). The median number of days of muscle strengthening activities increased by +2.0 days (95% CI 0.0 to +3.0) in the intervention group and did not increase in the control group (0.0 [95% CI 0.0 to +1.0], *P*=.06). In the intervention group, 52% (11/21) of participants reported having increased their muscle strengthening activities before surgery compared with 19% (3/16) of participants in the control group (*P*=.04; Figure 4).

#### Alcohol Consumption

Of the participants, 15% (12/79) had risk behavior regarding alcohol consumption, and 92% (11/12) of those completed the presurgery questionnaire (intervention group [2/11], control group [9/11]). Both participants in the intervention group (100%) reported a reduction in alcohol consumption before surgery compared with 11% (1/9) in the control group (*P*=.06; Figure 4).

#### Smoking

Of the participants 9% (7/79) were current smokers at baseline, and 43% (3/7) completed the follow-up questionnaire (intervention group [2/3] and control group [1/3]). In each group, 1 participant indicated having stopped smoking or smoked less in the run-up to surgery (Figure 4). Due to the small groups, no test was performed to compare groups.

#### Unintentional Weight Loss

Of the participants, 9% (7/79) had unintentionally lost 3 kg of weight in the last month. Due to an error in the online questionnaire, this outcome could not be evaluated.

### Functional Recovery

Of the participants, 81% (64/79) completed the questionnaire on physical functioning at baseline and 30 days after hospital discharge. At baseline, the median PROMIS-PF score was 47.8 (95% CI 43.9 to 60.1) in the intervention group (n=33) and 50.8 (95% CI 41.6 to 60.1) in the control group (n=31). Compared with baseline, both the intervention group (–6.2 [95% CI –10.6 to –2.1]) and the control group (–3.6 [95% CI –7.2 to 0.0]) had a lower level of physical functioning 30 days after discharge from hospital. Of the participants, 27% (9/40) in the intervention group and 35% (11/39) in the control group could be considered functionally recovered at 30 days after discharge from hospital. Using the complete cases, between-group analysis showed no meaningful difference in functional recovery after correction for baseline values (β=–2.4 [95% CI −5.9 to 1.1]).

## Discussion

### Principal Findings

The primary aim of this pilot study was to investigate the usability of the Be Prepared app prototype in patients undergoing major surgery. Both quantitative and qualitative data support the usability of the app and provide insight into adjustments that can be made to improve the app. Data from the SUS showed that patients considered the Be Prepared app to have acceptable usability (mean 68.2 [SD 18.4]) [25,27]. The app scored the best on the aspect of efficiency, the speed and ease with which the user gets something done [28,37]. Appreciation of the efficiency of the app is supported by our qualitative findings, as the majority of the interviewees found the app easy to use. The SUS score for learnability, the ease with which users accomplish basic tasks in the app for the first time [28], was above average. This was also supported by positive responses from the interviewees who found the app self-explanatory. Only the log-in procedure was described as difficult. The usability aspect of satisfaction had the highest and lowest SUS scores but could still be considered acceptable. The lowest scoring statement was “I think that I would like to use this app frequently,” which, in the context of the use of this app prior to surgery, is potentially confusing for participants. The major point of improvement suggested by the interviewees was further personalization of the app.

Our results suggest the Be Prepared app to be capable of bringing about a change in risk behaviors prior to major surgery. Participants who used the app became physically more active and increased their number of days of performing muscle strengthening activities. Furthermore, a larger proportion of app users indicated that they drank less alcohol in the run-up to their operation. No difference was found in smoking behavior between groups, but the sample size of both groups was too small to draw any conclusions. In our study, risk behavior has been assessed by means of online questionnaires. Although this applies to both groups, it may lead to socially desirable answers and perhaps an underestimation of risk behavior at baseline or an overestimation of the positive change in risk behavior. In spite of that, our findings are consistent with other research suggesting that individuals awaiting surgery welcome support to increase physical activities and reduce alcohol consumption but are less positive about smoking cessation [2].

Despite the positive trend in our intermediate outcome, change of risk behaviors, no preliminary effect of the Be Prepared app prototype on functional recovery 30 days after major surgery was found. The fact that we have not been able to show an effect on functional recovery after surgery can have several causes. Based on the concept that improving an individual’s functional capacity before major surgery helps them withstand the forthcoming stressor of major surgery, it makes sense to target those at high risk of postoperative complications and functional decline after surgery [4]. A recent systematic review describes that in many prehabilitation trials, inadequate patient selection may have led to an underestimation of the benefits of prehabilitation in patients undergoing major intra-abdominal surgery [38]. This is supported by the finding that only those studies targeting high-risk patients found significant improvements in physiological parameters and postoperative outcomes. In this pilot study, we included patients undergoing major surgery with an indication for postoperative hospital stay of a minimum of 2 nights and at least 1 risk behavior, with the aim to exclude low-risk patients. Nevertheless, participants in our pilot were on average younger and relatively fit in comparison with patients in other successful prehabilitation trials [38,39].

Furthermore, the Be Prepared app was a home-based unsupervised intervention. Evidence suggests that supervised prehabilitation may have a greater effect and higher adherence than unsupervised prehabilitation programs [4,38]. Offering prehabilitation by means of mHealth might not be sufficient for the entire group of surgical patients. Literature shows that especially patients with limited (digital) health literacy, advanced age, and chronic health conditions could benefit from extra support (blended care) in addition to mHealth [40-42]. Additional support by a health care provider during the preoperative period should therefore be considered for those patients in need of extra supervision.

### Limitations

This study is one of the first studies to explore the usability and preliminary effectiveness of an app for multimodal prehabilitation in patients undergoing major surgery. Our objective was to determine whether the Be Prepared app was usable and beneficial for a wide range of surgical patients. Therefore, our study sample was deliberately more heterogeneous than samples in other prehabilitation trials [38,39,43,44]. In hindsight, the 30-day follow-up may not have been the ideal time to identify an improvement in functional recovery for this diverse group of surgical patients, as the course of recovery is highly dependent on, among other things, the type of surgery [45]. Our results show that the majority of patients have not yet functionally recovered within 30 days of discharge from hospital. Future evaluation of the Be Prepared app will therefore include a longer follow-up with multiple time points to be able to compare (the course of) functional recovery between groups.

This study suffered from a large amount of missing data, especially at the presurgery follow-up. This can be explained, in part, by the timing of this measurement. Participants were invited to complete the presurgery follow-up questionnaire 3 days preoperatively, which might not have been an ideal time as patients have their minds on other things right before their surgery [2]. Changing the timing of this measurement to 1 week after discharge from hospital may help to reduce the amount of missing data when conducting the definitive trial.

In this study, 73% of the possible app users activated the app. The difficult log-in procedure and differences in patient (digital) health literacy may have contributed to the substantial number of nonusers in the intervention group [46]. The proportion of patients accessing the app at least once was comparable to that of other apps [47]. Altering the log-in procedure and providing support during installation and first use of the app could increase initial app use, but it is well known that user engagement decreases over time [48]. This is consistent with our qualitative data showing that patients who had access to the app for more than 4 weeks before surgery did not use the app during the entire preoperative period. Besides patients not feeling the urge to change until shortly before surgery, the limited days of preoperative content in the Be Prepared prototype (14 days) could have contributed to these patients stopping use of the app. Expanding the content for those with a longer preoperative period could help increase user engagement prior to surgery.

### Conclusions

Overall, the results of this pilot RCT demonstrate that the Be Prepared app prototype for patients undergoing major surgery has potential in terms of usability and changing risk behavior prior to major surgery. The app seems to fit the needs of patients preparing for major surgery. Several points of improvement for the app and study procedures have been identified, which supports the further development of the Be Prepared app and adjustment of study procedures before evaluating its effectiveness in a large multicenter RCT. These include adaptation of the timing of follow-up measurements, additional support by the physiotherapist during the preoperative period, and expanding the preoperative content of the Be Prepared app.

## Appendix

Multimedia Appendix 1 Overview of app content.

Multimedia Appendix 2 Topic list for semistructured interviews.

Multimedia Appendix 3 CONSORT-eHEALTH checklist (V 1.6.1).

## Abbreviations

CeHRes: Centre for eHealth Research
CONSORT: Consolidated Standards of Reporting Trials of Electronic and Mobile Health Applications and Online Telehealth
IQR: interquartile range
mHealth: mobile health
PROMIS-PF: Patient-Reported Outcomes Measurement Information System physical functioning 8-item short form
RCT: randomized controlled trial
SUS: System Usability Scale

## References

[1] StatLine—Operaties in het ziekenhuis, 1995-2010.

[2] McDonald S, Yates D, Durrand JW, Kothmann E, Sniehotta FF, Habgood A, Colling K, Hollingsworth A, Danjoux G (2019). Exploring patient attitudes to behaviour change before surgery to reduce peri-operative risk: preferences for short- vs. long-term behaviour change. Anaesthesia.

[3] Tew GA, Ayyash R, Durrand J, Danjoux GR (2018). Clinical guideline and recommendations on pre-operative exercise training in patients awaiting major non-cardiac surgery. Anaesthesia.

[4] Scheede-Bergdahl C, Minnella EM, Carli F (2019). Multi-modal prehabilitation: addressing the why, when, what, how, who and where next?. Anaesthesia.

[5] Thomsen T, Villebro N, Møller AM (2014). Interventions for preoperative smoking cessation. Cochrane Database Syst Rev.

[6] Dronkers JJ, Chorus AMJ, van Meeteren NLU, Hopman-Rock M (2013). The association of pre-operative physical fitness and physical activity with outcome after scheduled major abdominal surgery. Anaesthesia.

[7] Banugo P, Amoako D (2017). Prehabilitation. BJA Education.

[8] Cabilan CJ, Hines S, Munday J (2015). The effectiveness of prehabilitation or preoperative exercise for surgical patients: a systematic review. JBI Database System Rev Implement Rep.

[9] Li C, Carli F, Lee L, Charlebois P, Stein B, Liberman AS, Kaneva P, Augustin B, Wongyingsinn M, Gamsa A, Kim DJ, Vassiliou MC, Feldman LS (2013). Impact of a trimodal prehabilitation program on functional recovery after colorectal cancer surgery: a pilot study. Surg Endosc.

[10] Durrand J, Singh SJ, Danjoux G (2019). Prehabilitation. Clin Med (Lond).

[11] Lawson PJ, Flocke SA (2009). Teachable moments for health behavior change: a concept analysis. Patient Educ Couns.

[12] Ferreira V, Agnihotram RV, Bergdahl A, van Rooijen SJ, Awasthi R, Carli F, Scheede-Bergdahl C (2018). Maximizing patient adherence to prehabilitation: what do the patients say?. Support Care Cancer.

[13] Carli F, Scheede-Bergdahl C (2015). Prehabilitation to enhance perioperative care. Anesthesiol Clin.

[14] van der Meij E (2019). Improving Postoperative Recovery by eHealth [Thesis].

[15] Michie S, Carey RN, Johnston M, Rothman AJ, de Bruin M, Kelly MP, Connell LE (2018). From theory-inspired to theory-based interventions: a protocol for developing and testing a methodology for linking behaviour change techniques to theoretical mechanisms of action. Ann Behav Med.

[16] van Gemert-Pijnen EWC, Nijland N, van Limburg M, Ossebaard HC, Kelders SM, Eysenbach G, Seydel ER (2011). A holistic framework to improve the uptake and impact of eHealth technologies. J Med Internet Res.

[17] Maramba I, Chatterjee A, Newman C (2019). Methods of usability testing in the development of eHealth applications: a scoping review. Int J Med Inform.

[18] Eysenbach G (2011). CONSORT-EHEALTH: improving and standardizing evaluation reports of Web-based and mobile health interventions. J Med Internet Res.

[19] Schulz KF, Altman DG, Moher D (2010). CONSORT 2010 statement: updated guidelines for reporting parallel group randomised trials. BMJ.

[20] Neelemaat F, Kruizenga HM, de Vet HCW, Seidell JC, Butterman M, van Bokhorst-de van der Schueren MAE (2008). Screening malnutrition in hospital outpatients. Can the SNAQ malnutrition screening tool also be applied to this population?. Clin Nutr.

[21] Dutch dietary guidelines 2015.

[22] Health Council of the Netherlands Physical activity guidelines 2017.

[23] Bartlett YK, Sheeran P, Hawley MS (2014). Effective behaviour change techniques in smoking cessation interventions for people with chronic obstructive pulmonary disease: a meta-analysis. Br J Health Psychol.

[24] Systems and software engineering—Systems and software Quality Requirements and Evaluation (SQuaRE)—System and software quality models: ISO/IEC 25010:2011.

[25] Brooke J (1996). SUS: A 'Quick and Dirty' Usability Scale.

[26] Bangor A, Kortum PT, Miller JT (2008). An empirical evaluation of the System Usability Scale. Int J Human Comput Interact.

[27] Sauro J, Lewis J (2012). Quantifying the User Experience: Practical Statistics for User Research.

[28] Nielsen J (1994). Usability Engineering, 1st Edition.

[29] Cook KF, Jensen SE, Schalet BD, Beaumont JL, Amtmann D, Czajkowski S, Dewalt DA, Fries JF, Pilkonis PA, Reeve BB, Stone AA, Weinfurt KP, Cella D (2016). PROMIS measures of pain, fatigue, negative affect, physical function, and social function demonstrated clinical validity across a range of chronic conditions. J Clin Epidemiol.

[30] Schalet BD, Hays RD, Jensen SE, Beaumont JL, Fries JF, Cella D (2016). Validity of PROMIS physical function measured in diverse clinical samples. J Clin Epidemiol.

[31] Lee EC, Whitehead AL, Jacques RM, Julious SA (2014). The statistical interpretation of pilot trials: should significance thresholds be reconsidered?. BMC Med Res Methodol.

[32] Sauro J (2013). 10 Things to Know About the System Usability Scale (SUS). MeasuringU.

[33] Moltu C, Stefansen J, Svisdahl M, Veseth M (2012). Negotiating the coresearcher mandate—service users' experiences of doing collaborative research on mental health. Disabil Rehabil.

[34] Castleberry A, Nolen A (2018). Thematic analysis of qualitative research data: Is it as easy as it sounds?. Curr Pharm Teach Learn.

[35] Creswell J, Poth C (2018). Qualitative Inquiry and Research Design. 4th Edition.

[36] (2020). PHYSICAL FUNCTION—A brief guide to the PROMIS Physical Function instruments.

[37] Cockton G (2013). The Encyclopedia of Human-Computer Interaction. Chapter 15. Usability evaluation.

[38] Thomas G, Tahir MR, Bongers BC, Kallen VL, Slooter GD, van Meeteren NL (2019). Prehabilitation before major intra-abdominal cancer surgery: a systematic review of randomised controlled trials. Eur J Anaesthesiol.

[39] Sanchez-Lorente D, Navarro-Ripoll R, Guzman R, Moises J, Gimeno E, Boada M, Molins L (2018). Prehabilitation in thoracic surgery. J Thorac Dis.

[40] Smith B, Magnani JW (2019). New technologies, new disparities: the intersection of electronic health and digital health literacy. Int J Cardiol.

[41] de Vries HJ, Kloek CJJ, de Bakker DH, Dekker J, Bossen D, Veenhof C (2017). Determinants of adherence to the online component of a blended intervention for patients with hip and/or knee osteoarthritis: a mixed methods study embedded in the e-exercise trial. Telemed J E Health.

[42] Hermsen S, Moons J, Kerkhof P, Wiekens C, De Groot M (2017). Determinants for sustained use of an activity tracker: observational study. JMIR Mhealth Uhealth.

[43] Bolshinsky V, Li MH, Ismail H, Burbury K, Riedel B, Heriot A (2018). Multimodal prehabilitation programs as a bundle of care in gastrointestinal cancer surgery: a systematic review. Dis Colon Rectum.

[44] Furze G, Dumville JC, Miles JNV, Irvine K, Thompson DR, Lewin RJP (2009). “Prehabilitation” prior to CABG surgery improves physical functioning and depression. Int J Cardiol.

[45] Ljungqvist O, Francis N, Urman R (2020). Enhanced Recovery After Surgery: A Complete Guide to Optimizing Outcomes.

[46] Bol N, Helberger N, Weert JCM (2018). Differences in mobile health app use: a source of new digital inequalities?. Inf Soc.

[47] Lee M, Lee H, Kim Y, Kim J, Cho M, Jang J, Jang H (2018). Mobile app-based health promotion programs: a systematic review of the literature. Int J Environ Res Public Health.

[48] Buckingham SA, Williams AJ, Morrissey K, Price L, Harrison J (2019). Mobile health interventions to promote physical activity and reduce sedentary behaviour in the workplace: a systematic review. Digit Health.

