# Supplementary material for: Usability and Preliminary Effectiveness of a Preoperative mHealth App for People Undergoing Major Surgery: Pilot Randomized Controlled Trial
Source: JMIR Mhealth Uhealth. 2021 Jan 7;9(1):e23402. doi: 10.2196/23402 (PMC7819776; doi:10.2196/23402)
Supplement: Multimedia Appendix 1 [file mhealth_v9i1e23402_app1.pdf]

Appendix I: Overview of app content

Timeline	Topic	General description	Medium
		Surgery is like running a marathon	
		Risk behaviour screening questions<br>▪ currently smoking<br>▪ ≥ 1 alcohol consumption daily<br>▪ < 30 min moderate-intensity physical activity daily<br>▪ < 2 days a week muscle- strengthening activities<br>▪ ≥ 3kg unintentional weight loss in the last month	
		Patient experience with prehabilitation	
		Benefits of muscle strengthening before major surgery	
		Benefits of protein-rich food before major surgery	
		Benefits of smoking cessation	
		Motivation	
		How to increase your physical activity	
		Social support	
		Benefits of alcohol cessation	
		Effects of alcohol on anesthesia	
		Benefits of prehabilitation	
		Stair climbing	
		Benefits of combining exercise with protein-rich food.	
		Examples of protein rich food	
		The benefits of physical activity	
		Are you physically active for 30 minutes a day?	
		Smoking and taste	
		Smoking cessation support	
		When to consult a dietician	
		Alcohol cessation support.	
		When and what to eat	
		How to exercise	

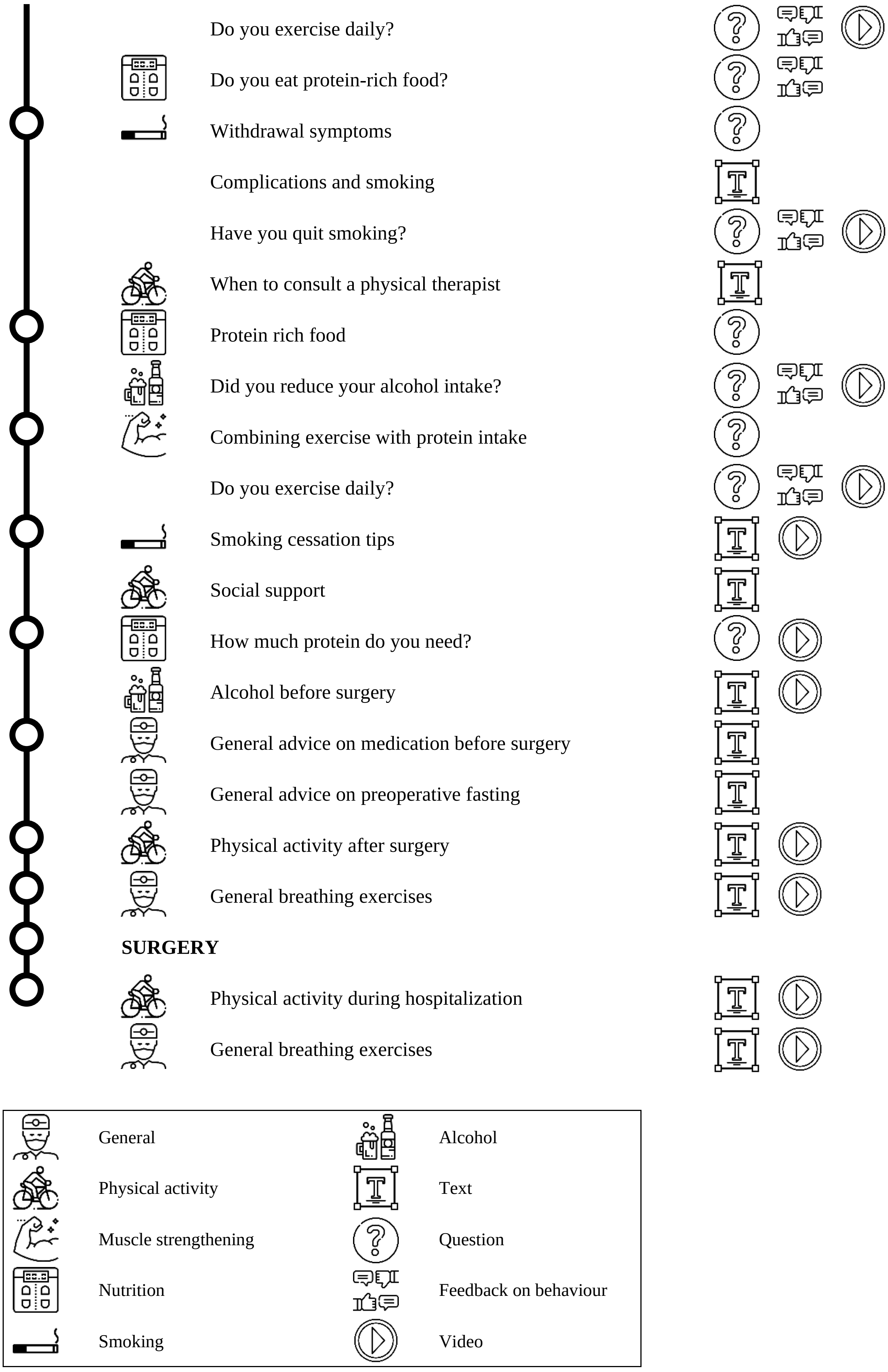

Do you exercise daily?
Do you eat protein-rich food?
Withdrawal symptoms
Complications and smoking
Have you quit smoking?
When to consult a physical therapist
Protein rich food
Did you reduce your alcohol intake?
Combining exercise with protein intake
Do you exercise daily?
Smoking cessation tips
Social support
How much protein do you need?
Alcohol before surgery
General advice on medication before surgery
General advice on preoperative fasting
Physical activity after surgery
General breathing exercises
SURGERY
Physical activity during hospitalization
General breathing exercises
General
Alcohol
Physical activity
Text
Muscle strengthening
Question
Nutrition
Feedback on behaviour
Smoking
Video
