# Supplementary material for: Usability and Preliminary Effectiveness of a Preoperative mHealth App for People Undergoing Major Surgery: Pilot Randomized Controlled Trial
Source: JMIR Mhealth Uhealth. 2021 Jan 7;9(1):e23402. doi: 10.2196/23402 (PMC7819776; doi:10.2196/23402)
Supplement: Multimedia Appendix 2 [file mhealth_v9i1e23402_app2.pdf]

Multimedia Appendix 2. Topic list for semistructured interviews.

Original topic list

1. Learnability
2. Memorability
3. Efficiency
4. Tolerance for errors
5. Satisfaction
6. Lifestyle changes

Topic list after first interview

1. Learnability
2. Memorability
3. Efficiency
4. Tolerance for errors
5. Satisfaction
6. Lifestyle changes
7. General experiences during preoperative and postoperative period
8. Preoperative and postoperative need for guidance

Topic list after fourth interview

1. Learnability
2. Memorability
3. Efficiency
4. Tolerance for errors
5. Satisfaction
6. Lifestyle changes
7. General experiences during preoperative and postoperative period
8. Preoperative and postoperative need for guidance
9. For postoperative interviews: Perceived effect of the lifestyle changes

Topic list after ninth interview

1. Learnability
2. Memorability
3. Efficiency
4. Tolerance for errors
5. Satisfaction
6. Lifestyle changes and motivation to change
7. General experiences during preoperative and postoperative period
8. Preoperative and postoperative need for guidance
9. For postoperative interviews: Perceived effect of the lifestyle changes
